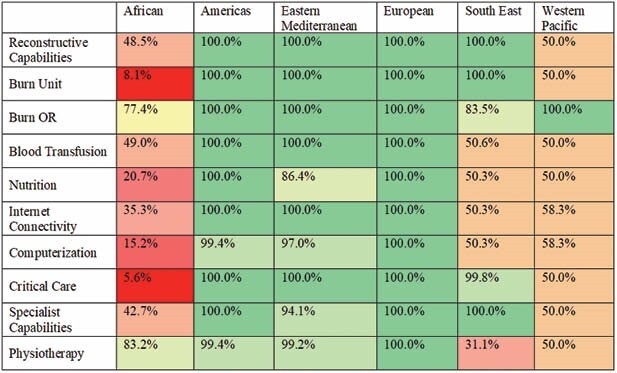# T2 Global Hospital Infrastructure and Pediatric Burns

**DOI:** 10.1093/jbcr/irac012.001

**Published:** 2022-03-23

**Authors:** Joseph S Puthumana, Charles S Hultman, Richard J Redett

**Affiliations:** Johns Hopkins University Department of Plastic and Reconstructive Surgery, Baltimore, Maryland; Johns Hopkins University School of Medicine, Baltimore, Maryland; Johns Hopkins University Department of Plastic and Reconstructive Surgery, Baltimore, Maryland

## Abstract

**Introduction:**

Low income regions across the world carry the highest proportion and highest mortality burden of pediatric burns. In recent years, attention to remedy these inequities has shifted from isolated mission trips towards building infrastructure for lasting improvements in surgical care. This study aims to investigate disparities in pediatric burn care infrastructure and their relationship to mortality outcomes.

**Methods:**

The multinational Global Burn Registry was queried for all burn cases between January 2018 and August 2021. Burn cases and mortality rates were compared across infrastructure factors by Pearson Chi-Square. Multinomial logistic regression was used to isolate independent modifiable risk factors for mortality controlling for total body surface area of burn (TBSA).

**Results:**

There were a total of 8537 cases of which 3492 (40.9%) were pediatric. Significantly lower mortality rates were found in facilities with sophisticated nutritional supplementation (p < 0.001), permanent internet connectivity (p < 0.001), reliable critical care access (p < 0.001), reliable burn OR access (p=0.003), dedicated burn unit (p < 0.001), and advanced plastic and reconstructive skills available (p=0.003); there was no significant difference if specialist capabilities in supportive fields (e.g. nephrology, cardiology) were available (p=0.063). Significant disparities were found in the availability of these resources between high and low income countries, as well granular information within low income regions: though blood transfusion access was limited in the African (49.0%) and South-East (50.6%) regions compared to full access in the Americas and Europe (p < 0.001), 100% of cases in the South-East had access to a specialized burn unit compared to 8.1% of cases in Africa. In a multinomial logistic regression controlling for TBSA, the most significant risk factor for mortality was limited critical care availability (OR 15.18, p< 0.001). The most significant protective factor was sophisticated nutritional access (OR 0.40, p=0.024).

**Conclusions:**

This is the first quantitative analysis of disparities in global burn infrastructure and their impact on pediatric mortality. The identification of nutritional support as an independent and significant protective factor suggests that low-cost interventions in hospital nutrition infrastructure merit further study and may realize significant gains in global burn care. Granular information in the variability of regional needs will begin to direct targeted infrastructure initiatives rather than a one-size-fits-all approach in developing nations.